# Supplementary material for: Asynchronous magnetic resonance elastography: Shear wave speed reconstruction using noise correlation of incoherent waves
Source: Magn Reson Med. 2022 Oct 27;89(3):990–1001. doi: 10.1002/mrm.29502 (PMC9792433; doi:10.1002/mrm.29502)
Supplement: Supplementary file 1 — DATA S1 MATLAB code used to generate the numerical simulations of Figure 1 [file MRM-89-990-s002.zip › k-Wave/helpfiles/roundEven.html]

roundEven :: Functions (k-Wave)


# roundEven

Round towards the nearest even number.

## Syntax

```
x = roundEven(x)
```

## Description

`roundEven` rounds each element of x towards the nearest even integer.

## Inputs

|  |  |
| --- | --- |
| `x` | input value/s |

## Outputs

|  |  |
| --- | --- |
| `x` | value/s of x rounded to nearest even integer |

## See Also

`round`, `roundOdd`
